# Birth characteristics and the risk of childhood leukaemias and lymphomas in New Zealand: a case-control study

**DOI:** 10.1186/1471-2326-6-5

**Published:** 2006-09-14

**Authors:** Donny IM Wong, John D Dockerty

**Affiliations:** 1 Department of Preventive and Social Medicine, University of Otago, Dunedin, New Zealand

## Abstract

**Background:**

Some studies have found that lower parity and higher or lower social class (depending on the study) are associated with increased risks of childhood acute lymphoblastic leukaemia (ALL). Such findings have led to suggestions that infection could play a role in the causation of this disease. An earlier New Zealand study found a protective effect of parental marriage on the risk of childhood ALL, and studies elsewhere have reported increased risks in relation to older parental ages. This study aimed to assess whether lower parity, lower social class, unmarried status and older parental ages increase the risk of childhood ALL (primarily). These variables were also assessed in relation to the risks of childhood acute non-lymphoblastic leukaemia, non-Hodgkin's lymphomas and Hodgkin's disease.

**Methods:**

A case control study was conducted. The cases were 585 children diagnosed with leukaemias or lymphomas throughout New Zealand over a 12 year period. The 585 age and sex matched controls were selected at random from birth records. Birth records from cases (via cancer registration record linkage) and from controls provided accurate data on maternal parity, social class derived from paternal occupation, maternal marital status, ages of both parents, and urban status based on the address on the birth certificate. Analysis was by conditional logistic regression.

**Results:**

There were no statistically significant associations overall between childhood ALL and parity of the mother, social class, unmarried maternal status, increasing parental ages (continuous analysis), or urban status. We also found no statistically significant associations between the risks of childhood acute non-lymphoblastic leukaemia, non-Hodgkin lymphomas, or Hodgkin's disease and the variables studied.

**Conclusion:**

This study showed no positive results though of reasonable size, and its record linkage design minimised bias. Descriptive studies (eg of time trends of ALL) show that environmental factors must be important for some diagnoses. Work has been done on the risk of ALL in relation to chemicals (eg pesticides) and drugs, dietary factors (eg vitamins), electromagnetic fields and infectious hypotheses (to name some); but whether these or other unknown factors are truly important remains to be seen.

## Background

Leukaemias are the most common cancers in childhood. Known causal factors for childhood leukaemias include genetic and familial factors, ionizing radiation, and cancer chemotherapy drugs, but together they account for a tiny proportion of cases. Two major hypotheses from Kinlen [1] and Greaves [2] have suggested that infections may be involved in the aetiology of childhood leukaemias. Feline leukaemia virus is well recognised as a cause of leukaemia in cats [3].

Infections in childhood may relate to demographic factors such as parity and social class. Reviews by Little [4], McNally et al. [5] and Poole et al. [6] have shown there have been many studies with inconsistent results concerning the roles that parity and social class play in childhood leukaemias. In a very large UK record linkage case-control study, there was a strong and significant protective effect of high parity on the risk of childhood acute lymphoblastic leukaemia (ALL) [7]. On the other hand, occupationally derived social class was not associated with the risk of childhood ALL. That study also showed that the risk of ALL increased significantly with increasing maternal and paternal ages [7].

In a smaller interview-based study in New Zealand, ALL risk was increased with parental unemployment (composite variable including both parents), low maternal education, and unmarried status of the mother at the time of diagnosis; but not with being firstborn [8]. The question therefore arose as to whether the findings from the interview-based New Zealand study were real, or a consequence of participation issues (92% participation among cases; 69% among controls) [8]. Birth record data from the eligible (rather than the participant) populations were also analysed in that study, and they showed that participation bias could not account for the unemployment and marital status findings [8].

A further question should also be asked: Are there differences in the way that variables such as these relate to childhood leukaemia risk in different countries, or do such differences in findings between countries occur simply as a result of sampling variation across studies? The current New Zealand study cannot answer this on its own. Nevertheless, it was proposed with the above questions in mind, to add to the international literature. While all childhood leukaemias and lymphomas were of interest, there was a particular focus on ALL, the commonest childhood cancer.

A record linkage case-control study design (involving cancer registrations and birth records) was used. This eliminated participation and recall bias. Birth records from cases and controls include information that can be used to classify children according to parity, social class (by paternal occupation) and marital status. Information in these records also allows an assessment of other potential risk factors – including parental ages and urban/non-urban status. The aim of this study was to look at whether these variables were risk factors for childhood leukaemias and lymphomas in New Zealand.

## Methods

In this study we sought to use additional data on the cases and controls who were in a previous New Zealand study of spatial clustering of leukaemias and lymphomas [9]. Ethical approval for this extension to the earlier study was granted soon after the clustering study was conducted. The cases were ascertained from the New Zealand Cancer Registry, being diagnosed in the period 1976–1987. We obtained the cancer registrations of all those diagnosed with leukaemia and lymphomas from throughout New Zealand. For leukaemias and non-Hodgkin's lymphomas (NHL) the age range was 0 to 14 years. The age range for Hodgkin's disease (HD) was extended to include all those aged from 0 to 24, as the disease has an older age distribution [10].

There were 748 children whose leukaemias and lymphomas had been registered in the relevant age ranges. Cases were only eligible for this study if they were born in New Zealand; therefore 76 of the 748 were excluded because they had another country of birth recorded on the cancer register. Twenty seven adopted children were also excluded. For 42 children, no birth records could be found, so they could not be included. Thus, we were left with 603 case group children who had relevant birth records [9]. In these 603 cases, there were seventeen cases of childhood leukaemias other than acute lymphoblastic leukaemia (ALL) and acute non-lymphoblastic leukaemia (ANLL), and these were excluded. One birth record of the remaining children was very incomplete on crucial variables, reducing the total number in the case group to 585.

A single age and sex matched control was randomly selected for each case from the New Zealand Birth Registry. The age matching was by quarter and year of birth registration. For example, if the birth of a case child was registered during the quarter 'January to March 1980', then the matched control was selected at random from the other children whose births were registered in the same quarter of the same year. Further details on the sample selection and record linkage are available elsewhere [9]. Birth certificate data were available for the cases (through the record linkage) and for their matched controls.

The following variables of interest were extracted or derived from the birth records: parity (no. of living or dead children within existing marriage), social class, marital status, parental ages, and urban/non-urban status. Social class was based upon the father's occupation using the Elley Irving scale. The Elley-Irving scale uses New Zealand Census data of median income and median education levels associated with each occupation for rating the occupation [11]. For marital status, those with no recorded marriage date were classified as unmarried and therefore they also had no parity data. The place of residence was classified as urban or non-urban, according to whether it lay within an urban or non-urban area as defined by the statistical boundaries used in the 1971 New Zealand Census [12].

Each diagnosis was analysed separately by conditional logistic regression, using STATA™ version 8.2. All the variables were considered as plausible confounders for each variable of interest, except certain relationships such as between parity and marital status, and between maternal and paternal ages. The variables were then tested with the data before finalising the list of confounders for the adjusted analyses. Generally, a variable was considered to be a confounder if it made a 10% (or greater) change in the odds ratio for the effect of a variable of interest, although social class was often included in the models (as in many previous studies). The confounders used in the ANLL and NHL analyses (which had smaller numbers) were the same as those identified for ALL.

For analysis of relevant variables as continuous data, we included the same confounder variables in the models that we had identified in the comparable categorical analyses. The results are presented as odds ratios (OR) with 95 % confidence intervals (CI).

## Results

In total, data for 585 cases and 585 controls were analysed. The cases comprised 278 children with ALL, 71 with ANLL, 79 NHL and 157 young people with Hodgkin's disease.

Table 1 presents the results of the conditional logistic regression for each diagnostic group. Increasing parity (as a continuous variable) was not associated with the risk of ALL (adjusted odds ratio (OR) 1.00 (95% confidence interval (CI) 0.85–1.16)), or with the other diagnoses assessed. The same lack of association was found for social class in relation to ALL (OR 0.97) and the other diagnoses. Marital status showed no significant association with risk of ALL (the adjusted OR for married vs unmarried was 1.5 (95% CI 0.9–2.6)); or with risk of the other diagnoses studied (Table 1). Urban status also showed no statistically significant results.

**Table 1:** Odds ratios for childhood leukaemias and lymphomas (conditional logistic regression)

**Variable**	**Categories**	Cases,Controls*	**Unadjusted OR (95% CI)**	**Adjusted OR (95% CI)**	Cases, Controls*	**Unadjusted OR (95% CI)**	**Adjusted OR (95% CI)**
**Leukaemias**			**Acute lymphoblastic leukaemia (ALL)**^†^			**Acute non-lymphoblastic leukaemia (ANLL)**^†^	
Parity	0^‡^	71,82	1.0	1.0	15,17	1.0	1.0
	1	74,61	1.4 (0.9–2.2)	1.4 (0.9–2.3)	11,16	0.7 (0.2–2.0)	0.8 (0.2–2.6)
	2	48,49	1.1 (0.7–1.9)	1.2 (0.7–2.0)	6,5	1.6 (0.4–6.2)	1.7 (0.4–7.8)
	≥ 3	22,23	1.1 (0.5–2.1)	1.1 (0.6–2.2)	14,8	2.7 (0.7–9.7)	2.1 (0.5–8.0)
		Continuous analysis^§ ^1.00 (0.85–1.16), p = 0.96			Continuous analysis^§ ^1.21 (0.93–1.58), p = 0.16		
Social class^||^	I or II	45,44	1.0 (0.6–1.7)	-	6,12	0.5 (0.1–1.7)	-
	III	79,66	1.2 (0.8–1.9)	-	16,19	0.6 (0.2–1.9)	-
	IV	58,71	0.8 (0.5–1.3)	-	22,13	1.7 (0.6–4.7)	-
	V or VI^‡^	74,75	1.0	-	19,19	1.0	-
		Continuous analysis^§ ^0.97 (0.86–1.09), p = 0.60			Continuous analysis^§ ^1.23 (0.92–1.64), p = 0.17		
Marital status	Married	250,241	1.3 (0.8–2.2)	1.5 (0.9–2.6)	55,59	0.7 (0.3–1.6)	0.8 (0.3–1.9)
	Unmarried^‡^	28,37	1.0	1.0	16,12	1.0	1.0
Father's age (years)	<25	58,51	1.1 (0.7–1.8)	1.2 (0.8–1.9)	18,14	1.4 (0.5–4.1)	1.5 (0.5–5.0)
	25–29^‡^	106,108	1.0	1.0	17,18	1.0	1.0
	30–34	59,50	1.2 (0.8–1.9)	1.2 (0.8–1.9)	14,21	0.7 (0.2–2.1)	0.8 (0.2–2.9)
	35–39	28,31	0.9 (0.5–1.7)	1.0 (0.6–1.9)	6,9	0.7 (0.2–2.9)	0.9 (0.2–4.5)
	40+	6,17	0.4 (0.1–1.0)	0.4 (0.1–0.9)	9,2	4.5 (0.8–25.0)	4.7 (0.8–29.0)
		Continuous analysis^§ ^0.97 (0.94–1.00), p = 0.10			Continuous analysis^§ ^1.03 (0.98–1.09), p = 0.23		
Mother's age (years)	<20	29,23	1.2 (0.7–2.2)	1.4 (0.7–2.8)	7,11	0.6 (0.2–2.2)	0.5 (0.1–2.0)
	20–24	91,107	0.8 (0.5–1.2)	0.8 (0.5–1.2)	24,17	2.4 (0.9–6.4)	3.7 (1.2–11.7)
	25–29^‡^	104,97	1.0	1.0	17,24	1.0	1.0
	30–34	41,34	1.2 (0.7–2.0)	1.1 (0.6–1.9)	14,14	1.5 (0.6–4.0)	1.8 (0.6–5.4)
	35+	13,17	0.7 (0.3–1.5)	0.6 (0.2–1.3)	9,5	3.3 (0.7–14.3)	2.6 (0.5–13.3)
		Continuous analysis^§ ^0.99 (0.96–1.03), p = 0.78			Continuous analysis^§ ^1.01 (0.95–1.08), p = 0.64		
Urban status	Urban	175,185	0.9 (0.6–1.2)	0.9 (0.7–1.3)	51,50	1.1 (0.5–2.1)	1.2 (0.6–2.7)
	Non-urban^‡^	101,91	1.0	1.0	20,21	1.0	1.0

**Lymphomas**			**Non-Hodgkin's lymphomas**^†^			Hodgkin's disease**	
Parity	0^‡^	27,23	1.0	1.0	47,38	1.0	-
	1	8,14	0.4 (0.1–1.3)	0.3 (0.1–1.2)	43,46	0.8 (0.4–1.4)	-
	2	15,11	1.0 (0.4–2.9)	1.2 (0.4–3.6)	29,34	0.7 (0.3–1.3)	-
	≥ 3	12,14	0.6 (0.2–1.9)	0.6 (0.2–1.8)	26,27	0.8 (0.4–1.6)	-
		Continuous analysis^§ ^0.95 (0.75–1.20), p = 0.67			Continuous analysis^§ ^0.95 (0.82–1.10), p = 0.50		
Social class^||^	I or II	12,14	0.8 (0.3–1.9)	-	25,17	1.6 (0.8–3.4)	1.4 (0.7–3.1)
	III	21,22	0.8 (0.3–2.1)	-	38,41	1.0 (0.6–1.9)	1.0 (0.6–1.9)
	IV	15,15	0.9 (0.4–2.2)	-	46,47	1.1 (0.6–1.9)	1.1 (0.6–2.0)
	V or VI^‡^	22,19	1.0	-	44,48	1.0	1.0
		Continuous analysis^§ ^1.06 (0.86–1.32), p = 0.58			Continuous analysis^§ ^0.92 (0.78–1.09), p = 0.35		
Marital status	Married	67,74	0.4 (0.1–1.2)	0.5 (0.2–1.7)	152,150	1.4 (0.4–4.4)	1.2 (0.4–4.0)
	Unmarried^‡^	12,5	1.0	1.0	5,7	1.0	1.0
Father's age (years)	<25	18,19	0.7 (0.3–1.6)	0.6 (0.2–1.4)	30,25	1.1 (0.6–2.2)	1.2 (0.6–2.5)
	25–29^‡^	27,19	1.0	1.0	53,50	1.0	1.0
	30–34	11,15	0.5 (0.2–1.3)	0.5 (0.2–1.5)	42,44	0.9 (0.5–1.6)	0.9 (0.5–1.7)
	35–39	10,11	0.6 (0.2–1.7)	0.6 (0.2–1.7)	15,16	0.9 (0.4–2.0)	0.7 (0.3–1.7)
	40+	5,7	0.5 (0.1–1.8)	0.5 (0.1–1.7)	13,18	0.7 (0.3–1.6)	0.6 (0.2–1.5)
		Continuous analysis^§ ^0.98 (0.93–1.02), p = 0.35			Continuous analysis^§ ^0.96 (0.93–1.00), p = 0.07		
Mother's age (years)	<20	13,7	1.4 (0.5–4.2)	0.9 (0.3–3.2)	6,12	0.5 (0.2–1.5)	0.9 (0.2–3.5)
	20–24	24,22	0.9 (0.4–1.9)	0.7 (0.3–1.7)	58,53	1.1 (0.7–1.9)	1.1 (0.6–2.0)
	25–29^‡^	25,22	1.0	1.0	50,50	1.0	1.0
	30–34	12,18	0.5 (0.2–1.4)	0.5 (0.2–1.3)	24,29	0.8 (0.5–1.6)	0.7 (0.3–1.4)
	35+	5,10	0.4 (0.1–1.4)	0.4 (0.1–1.3)	19,13	1.4 (0.6–3.4)	1.7 (0.6–4.5)
		Continuous analysis^§ ^0.97 (0.92–1.03), p = 0.37			Continuous analysis^§ ^0.99 (0.94–1.04), p = 0.69		
Urban status	Urban	47,54	0.7 (0.3–1.3)	0.6 (0.3–1.4)	94,96	0.9 (0.6–1.5)	0.9 (0.5–1.5)
	Non-urban^‡^	31,24	1.0	1.0	63,61	1.0	1.0

* The numbers are for cases and controls who contributed to the unadjusted analyses. In adjusted analyses, they vary depending on missing values in each variable in the model.

^† ^The confounders included for each variable are, (a) parity: social class; (b) social class: none; (c) marital status: mother's age; (d) father's age: social class; (e) mother's age: social class and, (f) urban status: social class.

^‡ ^Reference category.

^§ ^The result is the odds ratio that occurs as the variable increases by one unit, either by one year or one social class category (poorer) or one child, adjusted with confounders used in the categorical analyses.

^|| ^Category I represents those of highest socio-economic level, while VI the lowest socio-economic level.

** The confounders included for each variable are, (a) parity: none; (b) social class: mother's age; (c) marital status: mother's age; (d) father's age: social class & parity; (e) mother's age: social class & parity and, (f) urban status: social class & parity.

There were two results in the parental ages that were statistically significant at p < 0.05 and these were in the subcategories. For ALL, the oldest paternal age group (40+) showed a decreased risk in reference to age group 25–29, with an adjusted odds ratio of 0.4 (95% CI 0.1–0.9). Trend analysis of paternal age for ALL gave an odds ratio of 0.97, with a p value of 0.10. For ANLL, the second-youngest maternal age group (20–24) showed an increased risk in reference to age group 25–29, with an adjusted odds ratio of 3.7 (95% CI 1.2–11.7).

## Discussion

This national case-control study tested hypotheses relevant to a postulated infectious aetiology for childhood ALL, elucidating the roles of social class and parity. It also assessed other birth characteristics found to be associated with risk in other studies, such as increasing parental ages and unmarried status. No statistically significant associations were found overall, and the findings for the two subcategories for parental ages described above seem likely to be due to chance variation – they were not predicted *a priori*.

The record linkage design protected against some of the biases that were problematic in some earlier studies. These included those related to selection and participation issues, as well as potential recall bias in interview-based studies. By including young people with leukaemias and lymphomas who were diagnosed throughout New Zealand over a 12 year period, this study was the largest conducted so far in this country, and it had statistical power that was similar to or greater than in a good number of other studies.

A limitation in this study is the limited amount of information that was on New Zealand birth certificates, thus not permitting analysis of other potentially important perinatal factors, such as birth weight and maternal conception history. Parity derived in this study from the birth records is within an existing marriage. Fewer of those with Hodgkin's disease had missing parity data than was the case for those with the other diagnoses. This is because those with Hodgkin's disease were more likely to have been born in earlier years, and more of their mothers were of an older generation and married when the children were born.

No relationship between parity and childhood acute leukaemia was found in this study, similar to four other studies from Germany [13], Northern Ireland [14], Denmark [15] and California [16]. Another Californian birth record study looking specifically at the under 5 age group also did not find any relationship [17]. A study in the US [18] showed that higher parity was associated with an increased risk of childhood acute lymphoblastic leukaemia, while a very large record linkage study in England and Wales [7] showed a marked protective effect of increasing parity. The differences between the studies could be due to random variation or biases, or there may be other unknown (even non-infectious) variables that relate to cancer risk differently in different countries.

In our study, if a mother had previous live births in an earlier marriage or out of wedlock then her total parity would be underestimated. Some other previous studies that had additional information on these births might not be so directly comparable with ours.

No statistically significant association was found between ALL and social class based on parental occupation, similar to recent studies in England and Wales [7], Denmark [19] and Jerusalem [20].

A previous New Zealand interview based case-control study with smaller numbers showed that parental marriage significantly lowered the risk of ALL [8]. In contrast this record linkage study did not confirm this. The different findings may partly reflect differences in the rates of marriage and divorce over time. Unmarried parents were much less common in the present study; this study involves children diagnosed in an earlier (but longer) period.

Between the 1960s and 1990 there was a major increase in the incidence of ALL in children under five years of age in New Zealand [21]; similar increases have been seen in many other countries at different times. The increase was not an artefact of registration or of altered diagnostic classification, so it must represent a change in some unknown risk or protective factor. Work has been done on the risk of ALL in relation to chemicals (eg pesticides) and drugs, dietary factors (eg vitamins), electromagnetic fields and infectious hypotheses (to name some) [22]; but whether these or other unknown factors are truly important remains to be seen.

Hodgkin's disease was the second largest diagnostic group in this study, after ALL. However there were no statistically significant findings for this diagnosis. The HD cases studied here fit in better with a young adult population. The impact of factors acting around the time of birth (as assessed in this study) may therefore seem more questionable for HD than for the other childhood cancers which have a younger age distribution. However some studies of children and young adults have reported associations between correlates of higher social class and HD [23]. Higher social class [24,25] and low parity [26,27] have been shown to be associated with HD in the young adult. These factors could play a role in the link between Epstein-Barr virus infection and HD in the young adult population, because of the possible role of delayed infection as a risk factor [28,29], and the observation that an increased risk of HD persists for up to two decades after infectious mononucleosis [29]. However the epidemiology of Hodgkin's disease is complex, and involves the likely interplay of infections, the immune system and genetic susceptibility [23].

## Conclusion

The negative findings of this study concur with those of several others. Researchers should continue looking for the causes of childhood leukaemias and lymphomas, despite the relative lack of progress with the commonest childhood cancer, ALL. The most practical methodology we have for unravelling the causes of rare diseases is the case-control study. Although interview-based studies often suffer from biases due to control group selection, participation and recall issues, their ability to ask specific aetiological questions gives them an advantage over record-based studies. The latter are less subject to bias but they rely on existing data, so they can address a limited number of research questions. The challenge is to continue to make the methods of case-control studies as robust as possible, while pursuing existing leads and thinking of innovative new research questions to ask.

## List of abbreviations used

ALL, acute lymphoblastic leukaemia

NHL, non-Hodgkin's lymphoma

HD, Hodgkin's disease

ANLL, acute non-lymphoblastic leukaemia

OR, odds ratio

CI, confidence interval

## Competing interests

The authors declare that they have no competing interests.

## Authors' contributions

DIMW helped plan the analysis, conducted the analysis, interpreted the data and drafted the manuscript.

JDD conceived of and designed the study, collected the data, advised on data analysis and interpretation, and contributed to drafting the manuscript.

Both authors contributed edits following the peer review and approved the final manuscript.

## Pre-publication history

The pre-publication history for this paper can be accessed here:


